# A Voice from Lilliput

**Published:** 1867-12

**Authors:** 


### *A Voice from Lilliput.*

A city contemporary has come to the conclusion that "something more than bricks and mortar is necessary to make a Medical College." The Apostle insinuates in the remark his ideal addition—a manger with a dog in it.

The Apostle is further melancholy, as a eunuch at the door of a seraglio, over the burden of debt resting upon Rush Medical College. It is fortunate for the bantling which he is nursing that it is impossible for it to get into debt. But for the relief of our suffering neighbor, we suggest that if he can find on the market, in bank, or elsewhere, any certificates of the indebtedness of Rush Medical College, he had much better invest in them than mortages to the quondam ice-man who dismantled a lager-bier saloon to make the diminutive shell of a Medical College we wót of. However, as the Apostle naively stated in his opening address, it is unquestionably big enough for all who will seek its shelter.

The Faculty of Rush Medical College have erected and *paid* for their new edifice, not by levying contributions on their acquaintances, but with money from their own pockets. We opine this peculiar financial method is altogether inconceivable by the attaches of the "Reform" School, but the JOURNAL makes the statement for the benefit of such of its readers as may have noticed the late melancholy wail from the Apostolic organ.

